# Clinical Progression, Viral Load Dynamics, and Immune Kinetics of Nipah Virus Infection: Nipah Virus Kinetics Study Protocol

**DOI:** 10.1002/cpz1.70508

**Published:** 2026-10-24

**Authors:** Anika Farzin, Shadman Sakib Choudhury, Wasik Rahman Aquib, Sharmin Sultana, Mintu Chowdhury, Faruq Abdulla, Arifa Nazneen, Kamal Ibne Amin Chowdhury, Tonmoy Sarkar, Dewan Imtiaz Rahman, Mohammad Mamun Alam, Md. Mahfuzur Rahman, Ayesha Siddika, Tahsin Tabassum Anonto, Sinthia Karim, Md. Taufiqur Rahman Bhuiyan, Christina F. Spiropoulou, Michael K. Lo, Trevor Shoemaker, Mohammed Ziaur Rahman, Sayera Banu, Tahmina Shirin, Firdausi Qadri, Joel M. Montgomery, Syed Moinuddin Satter

**Affiliations:** ^1^ International Centre for Diarrhoeal Disease Research Bangladesh (icddr,b) Dhaka Bangladesh; ^2^ Institute of Epidemiology Disease Control and Research (IEDCR) Dhaka Bangladesh; ^3^ Viral Special Pathogens Branch, Division of High Consequence Pathogens and Pathology Centers for Disease Control and Prevention Atlanta GA

**Keywords:** Nipah virus, serum biomarker, viral load, immunokinetics

## Abstract

**Background**: Nipah virus (NiV) is a zoonotic pathogen with pandemic potential. Understanding the immune response to this pathogen is important for developing vaccines and therapeutics. This study aims to characterize viral load, host immune kinetics, and biomarker progression throughout NiV infection.

**Methods**: We will utilize the ongoing NiV surveillance platform in Bangladesh to enroll individuals with confirmed NiV infection. Oropharyngeal swabs and blood samples will be collected following the protocol. Oropharyngeal swabs will be taken daily until two consecutive negative results are obtained. Blood samples will be collected every 48 hours from initial detection of the virus until discharge from the hospital to assess IgM and IgG. Among survivors, 5 ml of blood will be collected every 15 days for 3 months from initial detection of the virus and then every 30 days up to 6 months. Viral load will be measured using RT‐qPCR. Immune kinetics will be assessed using quantitative enzyme‐linked immunosorbent assay (ELISA), and biomarkers will be analyzed via ELISA. Clinical progression, viral load, immune response, and biomarker patterns will be observed and documented.

**Discussion**: The study is expected to characterize the temporal trends of viral load and immune responses and examine their association with clinical progression, epidemiological indices, and disease outcome. Findings from this study could enhance understanding of the human immune response to NiV and inform diagnostic schedules and vaccine and therapeutics development. © 2026 The Author(s). *Current Protocols* published by Wiley Periodicals LLC.

## ADMINISTRATIVE INFORMATION

See Table 1. Note: the numbers in curly brackets in this protocol refer to SPIRIT checklist item numbers. The order of the items has been modified to group similar items (see http://www.equator‐network.org/reporting‐guidelines/spirit‐2013‐statement‐defining‐standard‐protocol‐items‐for‐clinical‐trials/).

**Table 1:** Administrative Information

Title {1}	Clinical Progression, Viral Load Dynamics, and Immune Kinetics of Nipah Virus Infection: Nipah Virus Kinetics Study Protocol
Trial registration {2a and 2b}.	This is an observational study, in which we are merely observing patients with confirmed Nipah virus infection and testing their viral loads, IgM, IgG, and serum biomarkers in different time frames to observe the clinical progression of Nipah virus infection. Please note that no experimental trial was conducted in this study.
Protocol version {3}	Protocol no.: PR‐22165, version 1.03, date 31.01.2023
Funding {4}	Centers for Disease Control and Prevention
Author details {5a}	1. Dr. Anika Farzin, International Centre for Diarrhoeal Disease Research, Bangladesh (icddr,b), Dhaka, Bangladesh 2. Dr. Shadman Sakib Choudhury, International Centre for Diarrhoeal Disease Research, Bangladesh (icddr,b), Dhaka, Bangladesh 3. Dr. Wasik Rahman Aquib, International Centre for Diarrhoeal Disease Research, Bangladesh (icddr,b), Dhaka, Bangladesh 4. Dr. Sharmin Sultana, Institute of Epidemiology, Disease Control and Research (IEDCR), Dhaka, Bangladesh 5. Dr. Mintu Chowdhury, Institute of Epidemiology, Disease Control and Research (IEDCR), Dhaka, Bangladesh 6. Mr. Faruq Abdulla, International Centre for Diarrhoeal Disease Research, Bangladesh (icddr,b), Dhaka, Bangladesh 7. Dr. Arifa Nazneen, International Centre for Diarrhoeal Disease Research, Bangladesh (icddr,b), Dhaka, Bangladesh 8. Mr. Kamal Ibne Amin Chowdhury, International Centre for Diarrhoeal Disease Research, Bangladesh (icddr,b), Dhaka, Bangladesh. 9. Mr. Tonmoy Sarkar, International Centre for Diarrhoeal Disease Research, Bangladesh (icddr,b), Dhaka, Bangladesh. 10. Dr. Dewan Imtiaz Rahman, International Centre for Diarrhoeal Disease Research, Bangladesh (icddr,b), Dhaka, Bangladesh 11. Dr. Mohammad Mamun Alam, International Centre for Diarrhoeal Disease Research, Bangladesh (icddr,b), Dhaka, Bangladesh. 12. Mr. Md. Mahfuzur Rahman, International Centre for Diarrhoeal Disease Research, Bangladesh (icddr,b), Dhaka, Bangladesh. 13. Miss Ayesha Siddika, International Centre for Diarrhoeal Disease Research, Bangladesh (icddr,b), Dhaka, Bangladesh. 14. Miss Tahsin Tabassum Anonto, International Centre for Diarrhoeal Disease Research, Bangladesh (icddr,b), Dhaka, Bangladesh. 15. Miss Sinthia Karim, International Centre for Diarrhoeal Disease Research, Bangladesh (icddr,b), Dhaka, Bangladesh 16. Dr. Md. Taufiqur Rahman Bhuiyan, International Centre for Diarrhoeal Disease Research, Bangladesh (icddr,b), Dhaka, Bangladesh 17. Dr. Christina F. Spiropoulou, Viral Special Pathogens Branch, Division of High Consequence Pathogens and Pathology, Centers for Disease Control and Prevention, Atlanta, GA 18. Dr. Michael K. Lo, Viral Special Pathogens Branch, Division of High Consequence Pathogens and Pathology, Centers for Disease Control and Prevention, Atlanta, GA 19. Dr. Trevor Shoemaker, Viral Special Pathogens Branch, Division of High Consequence Pathogens and Pathology, Centers for Disease Control and Prevention, Atlanta, GA 20. Dr. Mohammed Ziaur Rahman, International Centre for Diarrhoeal Disease Research, Bangladesh (icddr,b), Dhaka, Bangladesh 21. Dr. Sayera Banu, International Centre for Diarrhoeal Disease Research, Bangladesh (icddr,b), Dhaka, Bangladesh 22. Dr. Tahmina Shirin, Institute of Epidemiology, Disease Control and Research (IEDCR), Dhaka, Bangladesh 23. Dr. Firdausi Qadri, International Centre for Diarrhoeal Disease Research, Bangladesh (icddr,b), Dhaka, Bangladesh, and Viral Special Pathogens Branch, Division of High Consequence Pathogens and Pathology, Centers for Disease Control and Prevention, Atlanta, GA 24. Dr. Syed Moinuddin Satter, Corresponding Author, International Centre for Diarrhoeal Disease Research, Bangladesh (icddr,b), Dhaka, Bangladesh
Name and contact information for the trial sponsor {5b}	Centers for Disease Control and Prevention—Viral Special Pathogens Branch (VSPB), Atlanta, GA, USA
Role(s) of sponsor {5c}	Conceptualization, funding acquisition, writing—review and editing

## INTRODUCTION

### Background and Rationale {6a}

Nipah virus (NiV) was declared one of the top ten viruses with pandemic potential by the World Health Organization (WHO) in 2018 (Satter et al., 2023). This deadly virus causes severe pneumonitis and or encephalitis and has killed 40‐75% of its victims in different countries (Epstein et al., 2006). To date, NiV infections have been found in five countries in Southeast Asia (Sun et al., 2024). NiV infection was first detected as a respiratory and neurological disease in pigs and humans in Malaysia in 1998‐1999. The virus was then transmitted to Singapore and caused 276 human cases of encephalitis, leading to 106 deaths (Chua et al., 2000). The second outbreak occurred in India in 2001 (Chadha et al., 2006). Since 2001, Bangladesh has been the only country reporting active cases almost every year (Satter et al., 2023). The virus is usually shed through all body secretions of the bat, especially the saliva, urine, and excreta, which may contaminate raw date palm sap, fermented raw date palm sap (known as tari), and fruits half‐eaten by bats (Hughes et al., 2009; Luby et al., 2006). NiV can also be transmitted person to person through close contact with an infected patient's body fluids or by droplet infection (Clayton et al., 2016).

Even though it has been 25 years since the first known human NiV infection, there is a significant lack of scientific information due to its high fatality rate and the simple fact that only one country, Bangladesh, has systemic human surveillance covering most geographic areas to detect and confirm NiV cases. As the world has started to focus on developing vaccines and therapeutics against this deadly virus, information on the molecular level is greatly needed.

The human body's immune response to NiV is mediated by both innate and humoral immunity. The kinetics of IgM and IgG were observed in the serum of patients with NiV in a study conducted after the first outbreak in Malaysia by Ramasundpum et al., which described the kinetics of IgM and IgG against NiV but focused only on the quality of the test results (Ramasundpum et al., 2000). Wong et al. also elaborated on a late presentation of a confirmed case of NiV for which the IgG level was documented over a period of 2 years (Wong, 2001). Another study discussed the relationship between NiV and how immune agonists for it are provided by the body, which involves triggering of class I major histocompatibility complex (MHC) molecules and dendritic cells along with other cytokines (Seto et al., 2010). The evidence came from the first known outbreak of human NiV infection, and none of it described correlations with the clinical features of the cases. A study conducted in 2023 quantified immune cell populations and compared them between survivors up to 4 months after symptom onset and healthy controls (Sahay et al., 2024).

Another type of biological molecule that has a huge impact on immunity against viruses is the cytokines released by different cells of hosts in response to the infection (Chang et al., 2024). Recent studies show that cytokines have multifaceted interactions that are directly involved in the advancement of disease pathogenesis. Viruses induce the release of numerous cytokines that are activated by and also stimulate the expression or replication of extremely similar cytokines. Some cytokines, notably human interferons, are used in treating viral diseases (Sanapala & Pola, 2020). A study conducted on survivors and non‐survivors of COVID‐19 found that they showed significant differences in cytokine levels (Tan et al., 2020). Viral load is an aspect of viral infection that can be used to measure infection status and response to therapy (Glover & Dugas, 2016). Another study in COVID‐19 patients showed higher mortality among those with lower cycle threshold (Ct) values (denoting the number of cycles required to detect the SARS‐CoV‐19 virus by PCR, and thus inversely related to viral load), defined as Ct <30, than among those with higher Ct values (Ct ≥30) (*p* = .003; Shire et al., 2024). Another study, conducted in India, showed that NiV RNA could be detected in clinical samples till 14th day after symptom onset and when the viral load was in the range of 1.7‐3.3 × 10^4^ viral RNA copies/ml (Anoop Kumar et al., 2024).

As the world has started to focus on developing vaccines and therapeutics against this deadly virus, information on the molecular level is of high necessity. The proposed area of research is relatively unexplored; hence, limited relevant scientific information is available. This study is designed to increase understanding of the human immune response to NiV by exploring the evolution of immune kinetics (IgM and IgG) and key biomarkers (cytokines and chemokines) during infection. Clinical data will be generated to compare immune responses across NiV strains and high‐risk viruses by examining host‐virus interactions at the cellular and biochemical levels. These findings will aid the ongoing development of vaccines and therapeutics by providing a standard reference dataset. Correlation of clinical outcomes with viral load and immune kinetics may aid early prognosis and clinical management for future outbreaks. This study is based on existing surveillance infrastructure and has a strong capacity to advance the understanding of patient care and the development of therapeutics and vaccines. We propose to describe the clinical progression of NiV infection, determine the progression of viral load during NiV infection, and investigate host immune kinetics during and after NiV infection, as well as measuring serum biomarkers during and after NiV infection.

### Objectives {7}

1. To observe and document the clinical progression of NiV infection.
2. To determine the progression of viral load during NiV infection and the host immune kinetics during and after infection.
3. To measure the progression of serum biomarkers during and after NiV infection.

### Trial design {8}

We would like to mention that this study does not involve any experimental trial and is an observational study only.

## METHODS: PARTICIPANTS, INTERVENTIONS, AND OUTCOMES

### Study Design

This study will be conducted by leveraging the ongoing NiV surveillance platform, a plexus of thirteen active surveillance sites and three passive surveillance sites across Bangladesh. At each site, screening of suspected NiV cases will be conducted by surveillance staff. Data and samples will be collected from patients who meet the criteria of the suspected case definition and then processed. These samples will be transported back to Dhaka and tested routinely at the Institute of Epidemiology, Disease Control and Research (IEDCR) and the International Centre for Diarrhoeal Disease Research (icddr,b) to confirm the presence of NiV and of an immune response (IgM) against NiV. After confirmation of a NiV case, an outbreak response team will be formed. This group of experts will conduct a detailed investigation of the outbreak to explore the transmission and ensure prompt containment. Upon confirmation of NiV infection, oropharyngeal swabs will be collected every day, and blood will be collected every 48 hours, depending on the clinical conditions. This procedure will be strictly followed during the patient's hospitalization. Samples will be processed at the surveillance site (inside a biosafety hood, maintaining all necessary biosafety precautions) and shipped to icddr,b, Dhaka, for testing. Throat swabs will be tested to observe viral load, and serum will be tested to detect viral load and immune response (IgM). Ideally, the virus will become undetectable first in serum and then in a throat swab. When two successive throat swabs have tested negative by PCR, we will stop collecting swabs, and the patient will then be considered eligible for discharge from the point of view of viral transmission. We will keep collecting blood till the last day of hospitalization (to observe IgM and IgG). After discharge, we will collect blood samples every 15 days up to 3 months and then every 30 days up to 6 months to observe NiV IgG.

### Study Setting {9}

Starting in 2006, IEDCR and icddr,b have conducted surveillance on human NiV with technical support and funding from the U.S. Centers for Disease Control (Atlanta, GA, USA) and the Coalition for Epidemic Preparedness Innovations (CEPI). The surveillance has both sentinel and event‐based components. The event‐based components includes any reporting of NiV cases from any other sources than the hospital‐based NiV surveillance and enhanced NiV surveillance. This might be through phone call notification from any other hospitals rather than the tertiary hospitals where National Nipah Surveillance is established. There are currently sixteen sentinel surveillance sites (thirteen active and three passive), all of which are tertiary healthcare centers. These sites are evenly distributed across the administrative divisions of Bangladesh, covering almost the entire population of the country.

### Eligibility Criteria {10}

Only confirmed human NiV infections (confirmed through ELISA and/or PCR) detected by the active and passive NiV surveillance sites in Bangladesh (Fig. 1) and the event‐based platform will be used as sources of NiV cases for inclusion in this study.

**Figure 1:**
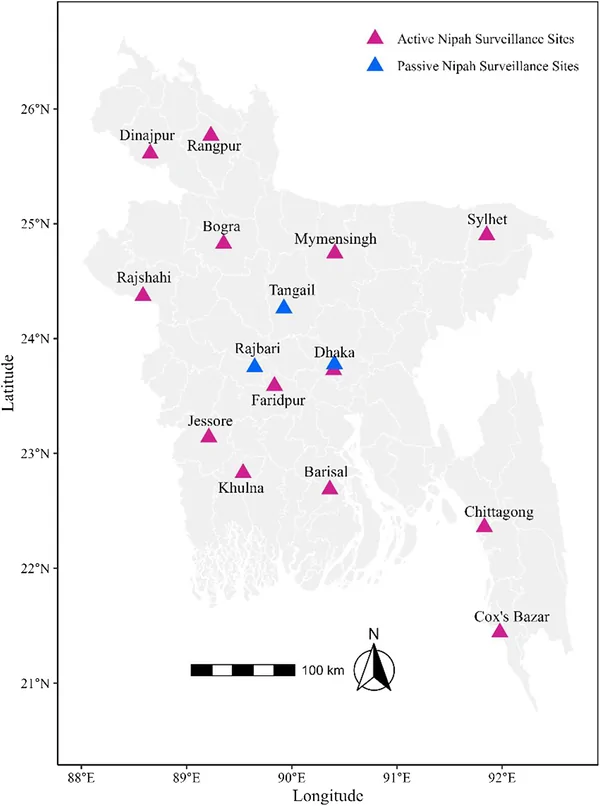
Sentinel sites of Nipah virus surveillance in Bangladesh.

### Who Will Take Informed Consent? {26a}

The surveillance physician of the respective ward of the respective tertiary medical college hospital site will collect written informed consent.

### Additional Consent Provisions for Collection and Use of Participant Data and Biological Specimens {26b}

This study design does not requires additional consent.

### Interventions

#### Explanation for the choice of comparators {6b}

This is a non‐interventional study.

#### Intervention description {11a}

This is a non‐interventional study.

#### Criteria for discontinuing or modifying allocated interventions {11b}

This is a non‐interventional study.

#### Strategies to improve adherence to interventions {11c}

This is a non‐interventional study.

#### Relevant concomitant care permitted or prohibited during the trial {11d}

This is a non‐interventional study.

#### Provisions for post‐trial care {30}

This is a non‐interventional study, so no post‐trial care is needed.

### Outcomes {12}

We have thoroughly reviewed the literature on different outcome variables and explanatory factors in our research. Based on our first objective, variables under the heading of “clinical progression” will consist of both numerical and categorical variables, and these will be the primary dependent variables. Primary independent variables will be the viral load, immune kinetics (IgM and IgG values against viral proteins), and chemokine levels, all of which will be numeric variables.

### Participant Timeline {13}

All confirmed NiV‐positive individuals identified at the sixteen surveillance sites from October 2022 to February 2027 will be approached and asked to enroll in this study for 6 months from the date of onset (Fig. 2).

**Figure 2:**
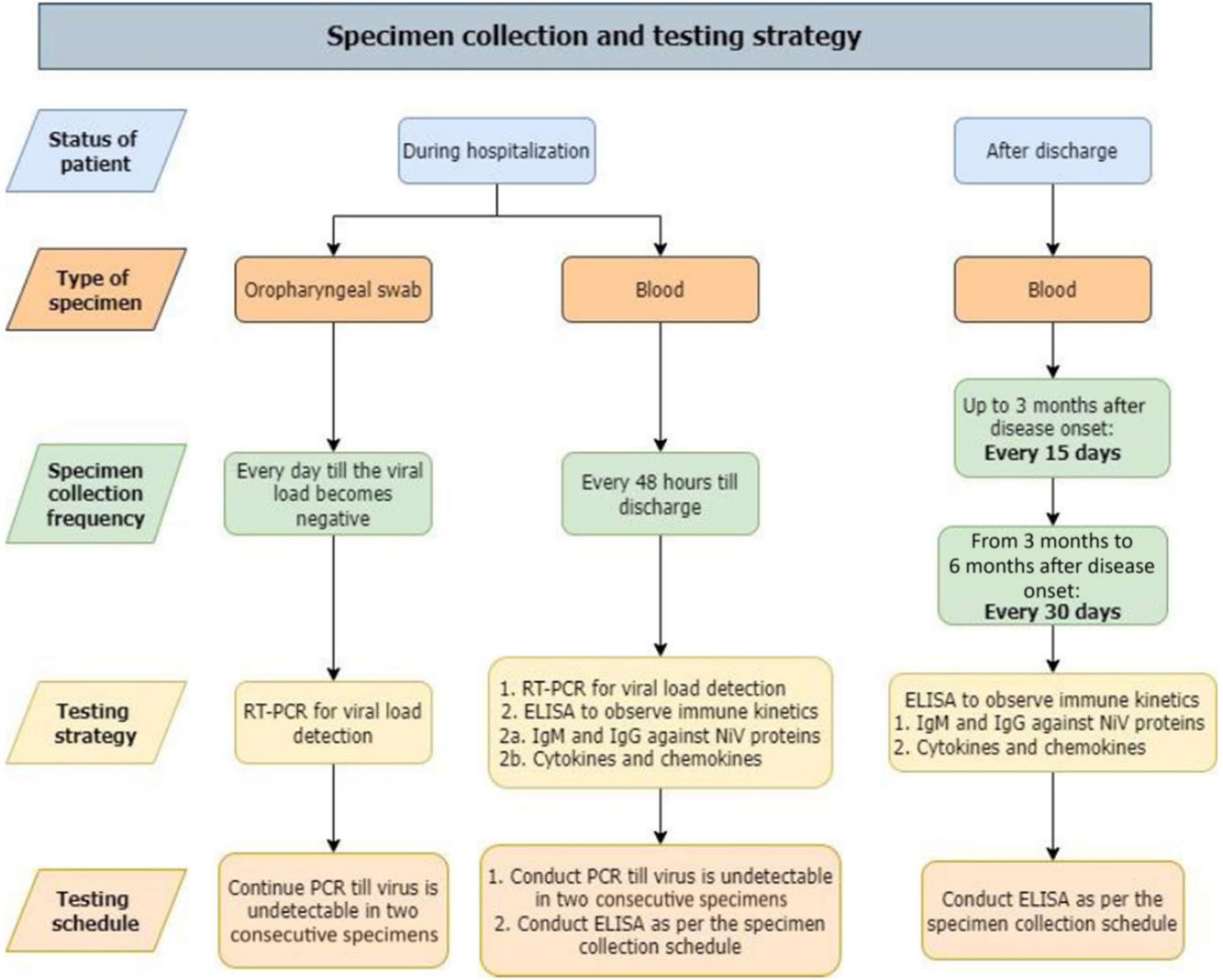
Specimen collection and testing strategy for observation of viral load and immune kinetics.

### Sample Size {14}

As the study's proposed activities are entirely based on the detection of NiV cases, the conventional methodology of sample size calculation does not apply here. Based on the data from ongoing surveillance, we have determined a tentative sample size: a total of 24 confirmed cases of NiV were detected in the past five years (2017‐2021), and therefore we anticipate detecting 12‐15 confirmed NiV cases during the study period of October 2022 to February 2027, all of whom will be approached for enrollment in this study. Here, it is important to clarify that NiV disease typically occurs during the winter season, particularly from December to April, and is associated with the consumption of raw date palm sap. For this reason, the NiV season is designated based on the year in which the majority of the season falls. We expect to enroll all future NiV infection survivors who contract NiV during the study period, including those who may not survive the infection.

### Recruitment {15}

Upon detecting a confirmed case of NiV, the patient will be enrolled upon providing consent, per the standard operating procedure for NiV surveillance. The surveillance physician will collect clinical information every 24‐48 hours, depending on the clinical conditions, following the clinical questionnaire of the NiV virus surveillance study (PR‐2005‐026). This documentation of clinical progression will cover a variety of physical and cognitive parameters, including fever, headache, respiratory symptoms, mental status, neurological abnormalities, and laboratory findings. The documentation schedule for NiV infection will include daily recordings of the patient's temperature, pulse, respiration rate, and other vital signs. In addition, weekly assessments will be conducted to monitor for changes in mental status. At ongoing surveillance sites, we have trained surveillance physicians in the medicine, pediatrics, and neurology departments. They work under the supervision of the focal persons, who are department heads of corresponding departments (basically professors in the respective departments who have postgraduate degrees). Moreover, as these healthcare facilities are tertiary‐level academic institutions, our surveillance physicians work in academic settings and are always supervised by trained, experienced clinicians and academicians. Most of our surveillance physicians have been involved in NiV surveillance activities for more than 3 years and have garnered substantial experience in the screening, physical examination, and recruitment of patients with suspected NiV. Additionally, we plan to organize a robust training workshop for surveillance physicians, which will be conducted by experienced neurologists and clinicians, to further strengthen their capacity. Under these circumstances, we will be able to monitor and document changes in the physical, mental status, and neurological abnormalities of the study participants.

### Assignment of Interventions: Allocation

#### Sequence generation {16a}

This is a non‐interventional study, so no sequence generation is needed.

#### Concealment mechanism {16b}

This is a non‐interventional study, so concealment is not necessary.

#### Implementation {16c}

This is a non‐interventional study, so nothing is implemented.

### Assignment of Interventions: Blinding

#### Who will be blinded {17a}

This is a non‐interventional study, so blinding is not needed.

#### Procedure for unblinding, if needed {17b}

This is a non‐interventional study, so unblinding is not required.

### Data Collection and Management

#### Plans for assessment and collection of outcomes {18a}

The NiV surveillance team will collect oropharyngeal swabs and blood from patients with NiV at regular intervals. Swabs will be collected every day until the viral load becomes undetectable in two consecutive samples, and 5 ml of blood will be collected every 48 hours until the patient is discharged. After patients are discharged, we will collect blood samples only (5 ml) at 15‐day intervals until 3 months after initial detection of the virus and then every 30 days till 6 months (Fig. 2). During hospitalization, samples will be collected in the hospital setting, and after discharge, sample collection and clinical evaluation will be done at the patient's residence.

#### Data management {19}

Data will be regularly checked for missing values, incorrect values, and invalid data.

#### Confidentiality {27}

Confidentiality of all the enrolled patients will be maintained as per icddr,b policy.

#### Plans for collection, laboratory evaluation, and storage of biological specimens for genetic or molecular analysis in this trial/future use {33}

##### Sample collection and transport

The NiV surveillance team will collect oropharyngeal swabs and blood samples at regular intervals. Swabs will be collected every day until the viral load becomes undetectable in two consecutive samples, and 5 ml of blood will be collected every 48 hours until the patient is discharged. After discharge, we will collect blood samples only (5 ml) at 15‐day intervals until 3 months after disease onset and then every 30 days until 6 months after disease onset (Fig. 2). During hospitalization, samples will be collected in the hospital setting, and after discharge, sample collection and clinical evaluation will be done at the patient's residence.

Samples will then be processed in the biosafety hood at the surveillance field office. The serum will be separated and stored in cryovials; one aliquot will be used for ELISA, and a second aliquot will be added to NucliSENS easyMAG Lysis Buffer in another cryovial for PCR. Swab samples will be stored in separate cryovials submerged in the same lysis buffer as well as viral transport medium (VTM). These cryovials will be shipped to the icddr,b laboratory in the Dhaka office using liquid‐nitrogen‐filled dry shippers. These samples will be stored at −80°C in the NiV surveillance freezer at the high‐containment laboratory of icddr,b. The processing, shipment, and storage of the clinical information and samples will be conducted leveraging the existing NiV surveillance workforce, logistics, and establishment.

##### Laboratory testing

Viral nucleic acid will be extracted from 200 µl of NiV‐positive serum sample collected in lysis buffer (NucliSENS easyMAG Lysis Buffer; bioMérieux) using InviMag Virus DNA/RNA Mini Kit (Invitek, STRATEC Molecular, Berlin‐Buch, Germany) on a Kingfisher Flex 96 (Thermo Fisher Scientific Inc.) automated nucleic acid extraction system according to the manufacturer's instructions. The nucleic acids will be eluted in 100 µl of elution buffer (Invitek, STRATEC Molecular, Berlin‐Buch, Germany) and stored at −80°C until used for PCR and sequencing tests. Initially, TaqMan PCR assay will be used to screen NiV RNA using NiV *N*‐gene‐specific primers and probe as described by Lo et al. (2012). The real‐time RT‐PCR assays will be carried out using the TaqPath 1‐Step Multiplex Master Mix kit (Thermo Fisher Scientific) with a final reaction volume of 20 µl, and CFX Opus Real‐Time PCR Systems (Bio‐Rad Laboratories, Inc., Hercules, CA, USA) operated by CFX Maestro Software version 1.1. The PCR master mix will contain 5 µl of 4× TaqPath 1‐Step Multiplex Master Mix, 0.5 µl of each primer (20 µM), 0.5 µl of the probe (10 µM), 8.5 µl of nuclease‐free water, and 5 µl of extracted RNA as template. The PCR reactions will be carried out under the following temperature cycling conditions: 25°C for 2 min in UNG incubation, 53°C for 10 min, 95°C for 2 min, and then 45 cycles of 95°C for 15 s, and 60°C for 1 min.

For viral load estimation, a plasmid control containing the NiV *N*‐gene sequence will be serially diluted (10‐fold dilutions ranging from 5.15 × 10^9^ copies/µl to 5.15 × 10^1^ copies/µl). This dilution series will serve as a positive control and standard curve for RT‐qPCR runs. During each RT‐qPCR run, the serially diluted standards (in triplicate) and the clinical samples (in duplicate) will be tested under identical conditions. A standard curve will be constructed using the log‐transformed copy number for each standard dilution. The average Ct value for each dilution, including both the plasmid control and clinical samples, will be determined, enabling calculation of the RNA copy number per microliter of input template.

The in‐house indirect NiV glycoprotein G IgG ELISA will be developed and qualified in accordance with the International Council for Harmonisation of Technical Requirements for Pharmaceuticals for Human Use (ICH) Q2(R2) guideline, and the full qualification dataset will be reported separately (Alam et al., 2026). For each plate, the cutoff will be calculated as twice the sum of the mean optical density of the blank wells and its standard deviation; samples at or above the cutoff will be reported as positive, and those below it as negative. Every run will include the WHO International Standard for anti‐NiV antibodies (NV‐1, National Institute for Biological Standards and Control [NIBSC] code 22/130_BA; 1000 IU/ml) as the calibrating standard, together with a characterized pooled convalescent positive control, a known negative human serum, and diluent‐only blank wells. IgG concentrations (in IU/ml) will be assigned by parallel‐line calibration against NV‐1 and therefore traceable to the international standard.

In qualification, the IgG assay correctly classified ten of ten reference and convalescent positive sera and nine of nine negative sera, giving 100% sensitivity (95% CI 94.96%‐100%) and 100% specificity (95% CI 87.51%‐100%); the confidence intervals reflect the small characterized panel available for a Biosafety Level 4 (BSL‐4) pathogen. Intra‐assay precision, assessed by two analysts in duplicate across three runs on separate days, gave geometric mean coefficients of variation of 1.73% and 4.31%, and inter‐assay precision was 4.16%, all within the pre‐specified limit of 25%. The assay was linear from 31.9 to 676.3 IU/ml (*R* ^2^ = 0.9964, slope 0.9738), relative accuracy was 95.05% with individual recoveries of 82%‐100% against an acceptance range of 80%‐120%, and the lower limits of detection and quantification were 0.51 and 0.98 IU/ml, respectively, with upper limits of 43.65 and 32.65 IU/ml at a maximum 24‐fold dilution.

The in‐house NiV IgM ELISA remains under development and has not been qualified. No WHO International Standard or other certified reference material exists for anti‐NiV IgM: the NIBSC preparation used for IgG is convalescent‐phase material with an assigned IgG value only, and no acute‐phase, IgM‐positive NiV panel is distributed internationally. IgM results will therefore not be expressed in international units. They will instead be reported qualitatively against the same plate‐wise cutoff, and semi‐quantitatively as endpoint titers from the twofold dilution series, normalized to a pooled acute‐phase in‐house reference serum from RT‐PCR‐confirmed cases that will be assigned an arbitrary unit value and run as a dilution series on every plate alongside a negative pool and diluent blanks. These titers are internally consistent but not transferable between laboratories. Qualification of the IgM assay under ICH Q2(R2) for precision, dilutional linearity, limits of detection and quantification, and analytical specificity is in progress and will be reported separately; absolute accuracy and traceability cannot be established until a reference standard for anti‐NiV IgM becomes available.

Analytical specificity of the IgG assay will be addressed during development by replacing an Fc‐tagged coating antigen that gives false‐positive reactions with known negative sera, and by selecting the coating concentration, conjugate dilution, and substrate incubation time that minimize nonspecific binding. Cross‐reactivity testing against related henipaviruses, particularly Hendra virus, and against sera from other endemic febrile illnesses has not been performed for either isotype and is planned as part of the continuing validation of this platform.

The concentrations of the cytokines interleukin (IL)‐10 (Thermo Fisher Scientific, USA), IL‐17A (Thermo Fisher Scientific, USA), and interferon (IFN)‐γ (Abbexa, USA) in serum specimens will be determined using sandwich ELISA, following the manufacturer's instructions. ELISA plates will be coated overnight with monoclonal antibodies specific to each cytokine, as provided in the respective kits. Serum samples will be diluted 1:10 with diluent buffer and applied to the coated plates.

A standard solution will be prepared by reconstituting the lyophilized standard in assay buffer (phosphate‐buffered saline containing 1% Tween 20 and 10% bovine serum albumin). This standard will then be serially diluted twofold across 8 consecutive wells in duplicate columns. Optical density (OD) will be measured at 450 nm using an ELISA reader (BioTek). A standard curve will be generated from the OD values, which will be used to extrapolate cytokine concentrations in the serum samples.

The sample testing schedule will coincide with the sample collection schedule, as mentioned in Figure 2. Viral load of NiV and kinetics of humoral immune particles, cytokines, and chemokines will be observed in the laboratory of icddr,b. We plan to send a copy of each specimen to the BSL‐4 laboratory at the CDC, Atlanta, which is the reference laboratory for activities on NiV. This is to ensure recovery and characterization of the virus, as well as quality control of the tests conducted at icddr,b laboratory.

### Statistical Methods

#### Statistical methods for primary and secondary outcomes {20a}

We will conduct descriptive analysis to summarize the demographic and all other characteristics of the participants. We will summarize the categorical variables using frequency and percentage. Fisher's exact test will be performed to compare proportions of categorical variables among groups. Viral load data, as well as antibody and cytokine responses data, will be log‐transformed before analysis. These data from the acute to convalescent phases among survivors will be analyzed using suitable graphical techniques to illustrate their rise, peak, and decline patterns throughout illness progression. Trend in viral load, antibody, and cytokine responses over the course of illness will be examined using a generalized additive model (GAM), and their interrelationship will be explored through overlaid time‐series plots. Viral load pattern will be compared between survivors and individuals with fatal cases of NiV using graphical visualizations.

The continuous measurements will be summarized using median and interquartile range (IQR). Statistical test for continuous measurements will include the Wilcoxon rank‐sum test to compare medians between different groups. In addition, the Wilcoxon signed‐rank test will be used to assess differences in continuous variables between two time points within the same group, and the Friedman test will be considered for comparisons across multiple time points.

#### Interim analyses {21b}

As we are only observing individuals with confirmed cases of NiV and performing tests using their samples, interim analysis is not required

#### Methods for additional analyses (e.g., subgroup analyses) {20b}

Not required for this study.

#### Methods in analysis to handle protocol nonadherence and any statistical methods to handle missing data {20c}

Not required for this study.

#### Plans to give access to the full protocol, participant level‐data and statistical code {31c}

Available upon request to the corresponding author.

### Oversight and Monitoring

#### Composition of the coordinating center and trial steering committee {5d}

Not required for this study.

#### Composition of the data monitoring committee, its role and reporting structure {21a}

Not required for this study.

#### Adverse event reporting and harms {22}

Not required for this study.

#### Frequency and plans for auditing trial conduct {23}

Not required for this study.

#### Plans for communicating important protocol amendments to relevant parties (e.g., trial participants, ethical committees) {25}

Not required for this study.

#### Dissemination plans {31a}

Not required for this study.

## DISCUSSION

Understanding the immune kinetics of NiV infection is critical for advancing the development of vaccines and therapeutics against this highly pathogenic virus. Due to its high fatality rate and limited availability of systematic surveillance, scientific evidence on clinical progression and host immune responses to NiV is scarce. This study is designed to address these knowledge gaps by documenting the temporal evolution of viral load, antibody responses (IgM and IgG), and serum biomarkers (cytokines and chemokines) in human NiV infection. The novelty of the proposed work is that it methodically links clinical progression with viral dynamics and immune markers at the individual level, an approach that has not been previously applied to this pathogen. By generating data on host immune responses, this study will not only provide innovative insights into the pathogenesis of NiV but also establish reference data that can contribute to vaccine design, therapeutic development, and clinical management during future outbreaks.

The pattern of viral load in consecutive samples will help clarify how long a patient's secretions may be infective to caregivers or health care professionals and assess the period of transmissibility of NiV. This knowledge about viral load may help us to understand the transmission patterns of different strains of NiV and also of other, similar viral diseases. In a study conducted in 2023, a computational model simulated a disease transmission process in which the viral load of the patient at the time of transmission affects how infectious the newly infected individual becomes. These transmission pair correlations lead to a population‐wide convergence, where the distribution of initial viral loads in each new generation gradually stabilizes into a steady‐state pattern (Harris et al., 2023).

Immunoglobulins are central elements of the adaptive response (Delves, 2020). Thus, their involvement in neutralizing a virus is of great significance for vaccine efficacy. Studies of NiV‐specific antibody responses have shown that neutralizing antibodies targeting the viral G and F glycoproteins can block viral entry and spread (Chen et al., 2024). Hence, elucidating the kinetics and specificity of this immunoglobulin response helps in vaccine design, against which optimal protection is needed. One study described a late presentation of a confirmed case of NiV in which the IgG level was documented over a period of 2 years (Wong, 2001). In other viral respiratory diseases, expression of IgG may take 4 weeks to develop and remain high for a year after exposure (Yan et al., 2024).

Several limitations of the serological methods should be acknowledged. The IgG ELISA is qualified and calibrated against the WHO International Standard for anti‐NiV antibodies, but the companion IgM assay is still under development, and no international reference material for anti‐NiV IgM exists; IgM responses will therefore be described qualitatively and as relative endpoint titers rather than in international units, and the IgM findings should be read as exploratory. In addition, neither assay has been tested for cross‐reactivity with closely related henipaviruses such as Hendra virus, or with sera from the other febrile illnesses endemic in this setting, a consideration of particular weight for acute‐phase IgM given the dengue, chikungunya, and Japanese encephalitis IgM responses common in this population. Binding antibody concentrations measured by ELISA also do not necessarily reflect neutralizing activity.

Biomarkers such as cytokines and chemokines provide the innate and inflammatory responses against NiV disease. Their patterns of expression can therefore influence the course of the disease (Turner et al., 2014). Previous studies have portrayed the excessive production of pro‐inflammatory cytokines such as tumor necrosis factor (TNF)‐α, IL‐6, and IFN‐γ as being associated with immunopathology and worsened outcomes in NiV‐infected individuals (Prescott et al., 2012). Equally, type I interferons play a protective role in the initial stages of infection by restricting viral replication (Teijaro, 2016). Through planning of these cytokine profiles in infected patients, researchers can identify markers of protective versus harmful responses. This information is vital not just for vaccine development but also for the development of therapeutics intended to modulate the immune system—by either enhancing antiviral responses or reducing excessive inflammation.

Chemokines—secreted from neutrophils, T cells, macrophages, mast cells, and eosinophils and are responsible for recruiting immune cells to the site of infection—are also involved in the progression of NiV‐related encephalitis and respiratory diseases. A disharmony in the regulation of response created by chemokine can lead to tissue damage through the exaggerated accumulation of immune cells at the inflammation site (Chen et al., 2017). Knowledge about which chemokines are upregulated during infection and how they correlate with disease progression can help identify targets for adjunct therapies to reduce immune‐mediated damage.

Altogether, the interaction between immunoglobulins, cytokines, and chemokines forms a complex immune response that determines the outcome of NiV infection. Analyzing these components will facilitate tailoring the vaccine platform, determining which kind of vaccine will work best, and designing therapeutics. Without this foundational knowledge, attempts to control NiV through immunization or treatment may fall short or cause unintended immune‐related complications.

Though there are studies describing the immune correlate of protection from NiV in nonhuman primates (Leyva‐Grado et al., 2024), this remains unexplored in infected human subjects. Therefore, this protocol will advance the field of NiV research by providing numerous pieces of information regarding immune responses against NiV.

### Strength and Limitations

Through this protocol, we will test biological samples of NiV survivors from 2022 to 2027, providing information regarding viral load, immune kinetics, and patients’ viral loads during different stages of the disease. This study will also gather information about cytokines and chemokines during NiV disease. A limitation of this study is that it will be applied only to NiV survivors, and because this is a rare and fatal disease, the sample size may be small.

## TRIAL STATUS

Please note that this is an observational study and does not include any trial‐based activity.

## DECLARATIONS

### Author Contributions

**Anika Farzin**: Methodology, visualization, writing—original draft, writing—review and editing. **Shadman Sakib Choudhury**: Project administration, supervision, writing—original draft, writing—review and editing. **Wasik Rahman Aquib**: Conceptualization, writing—review and editing. **Sharmin Sultana**: Writing—review and editing. **Mintu Chowdhury**: Writing—review and editing. **Faruq Abdulla**: Validation, writing—review and editing. **Arifa Nazneen**: Writing—review and editing. **Kamal Ibne Amin Chowdhury**: Writing—review and editing. **Tonmoy Sarkar**: Writing—review and editing. **Dewan Imtiaz Rahman**: Writing—review and editing. **Mohammad Mamun Alam**: Writing—review and editing. **Md. Mahfuzur Rahman**: Writing—review and editing. **Ayesha Siddika**: Writing—review and editing. **Tahsin Tabassum Anonto**: Writing—review and editing. **Sinthia Karim**: Writing—review and editing. **Md. Taufiqur Rahman Bhuiyan**: Conceptualization, writing—review and editing. **Christina F. Spiropoulou**: Conceptualization, funding acquisition, writing—review and editing. **Michael K Lo**: Funding acquisition, writing—review and editing. **Trevor Shoemaker**: Funding acquisition, writing—review and editing. **Mohammed Ziaur Rahman**: Conceptualization, visualization, writing—review and editing. **Sayera Banu**: Writing—review and editing. **Tahmina Shirin**: Writing—review and editing. **Firdausi Qadri**: Writing—review and editing. **Joel M. Montgomery**: Conceptualization, funding acquisition, writing—review and editing. **Syed Moinuddin Satter**: Conceptualization, methodology, project administration, visualization, resources, supervision, writing—original draft, writing—review and editing.

### Funding {4}

The study was funded by the US Centers for Disease Control and Prevention (CDC), Atlanta (https://www.cdc.gov/).

### Availability of Data and Materials {29}

The data used in this study can be made available upon reasonable request to the corresponding author.

### Ethics Approval and Consent to Participate {24}

Informed written consent will be obtained from all the participants as well as patient attendants. The protocol will be reviewed and approved by the Ethical Review Committee (ERC) of icddr,b.

### Consent for Publication {32}

Consent for publication from patients will be collected during patient enrollment and publication clearance has been obtained from icddr,b research administration.

### Competing Interests {28}

The authors declare no competing interests.
